# Impact of Tricuspid Repair on Surgical Death in Patients Undergoing Mitral Valve Surgery Due to Rheumatic Disease

**DOI:** 10.1016/j.shj.2024.100298

**Published:** 2024-04-04

**Authors:** Gustavo P. Santana, Rodrigo M. Vieira de Melo, Tainá T. Viana, Daniela N. Velame da Silva, Clara S. Figueiredo, Diogo F.C. de Azevedo, Osvaldemar R.N. Junior, Edmundo J.N. Câmara, Luanna M. Damasceno, Ana Luísa A.A. Silva, João Pedro M.M. Granja, Luiz C.S. Passos

**Affiliations:** a Ana Nery Hospital, Cardiology Department, Salvador-BA, Brazil; b Federal University of Bahia, Salvador-BA, Brazil

**Keywords:** Rheumatic valve disease, Tricuspid regurgitation, Tricuspid repair

## Abstract

**Background:**

Tricuspid valve repair during mitral valve replacement surgery remains a controversial topic. The risk-benefit ratio in some populations remains uncertain, especially in rheumatic heart disease patients. Therefore, we aimed to evaluate the impact of concomitant tricuspid repair on surgical mortality in patients undergoing cardiac surgery due to rheumatic mitral valve disease who have moderate to severe functional tricuspid regurgitation.

**Methods:**

This is a prospective cohort study from January 1, 2017, to December 30, 2022. All patients over 18 years of age who underwent cardiac surgery to correct rheumatic mitral valve disease with concomitant moderate to severe tricuspid regurgitation were included. The primary outcome was a surgical death. In an exploratory analysis, clinical and echocardiographic data were obtained 2 years after the procedure.

**Results:**

Of the 144 patients included, 83 (57.6%) underwent tricuspid valve repair. The mean age was 46.2 (±12.3) years with 107 (74.3%) female individuals, the median left ventricular ejection fraction was 61.0% (55-67), and systolic pulmonary artery pressure (sPAP) was 55.0 mmHg (46-74), with 45 (31.3%) individuals with right ventricular dysfunction. The total in-hospital mortality was 15 (10.4%) individuals, and there was no difference between the groups submitted or not to tricuspid repair: 10 (12.0%) vs. 5 (7.5%); *p* = 0.46, respectively. There was an association with one variable independently: the sPAP value, relative risk 1.04 (1.01-1.07), *p* = 0.01. The estimated cut-off value of sPAP that indicates higher early mortality through the receiver operating characteristic curve (area 0.70, *p* = 0.012) was 73.5 mmHg.

**Conclusions:**

Performing tricuspid repair in individuals who were undergoing cardiac surgery to correct rheumatic mitral valve disease was not associated with increased surgical mortality. Our results suggest the safety of tricuspid repair even in this high-risk population, reinforcing the recommendations in current guidelines.

## Introduction

Rheumatic heart disease is still a health problem worldwide, having high prevalence and morbidity in low- and middle-income countries but maintaining importance in specific populations in high-income countries, where it amounts to a third of concomitant valve diseases.^1^ Data from the Global Burden of Disease estimates that there were 3,604,800 cases of rheumatic heart disease on the American continents in 2017, with 22,437 deaths, representing an important socioeconomic impact.^2^

The valves most frequently involved in rheumatic heart disease are mitral and aortic; however, the tricuspid valve is commonly affected due to annular dilatation.^3^ Functional tricuspid regurgitation (TR) tends to progress when isolated treatment of primary valve disease is performed, especially when there is dilation of the tricuspid ring.^4^, ^5^, ^6^ Thus, the main guidelines recommend that TR be treated as part of the surgical procedure for left valve disease; however, this recommendation is based on small observational studies in which patients with rheumatic heart disease were underrepresented.^1^^,^^7^

In addition, some of these observational studies show an increase in mortality from annuloplasty related to the right ventricle’s dysfunction and circulatory shock in the immediate postoperative period.^8^ This data is particularly relevant for patients who are operated on late in advanced stages of heart disease, which is common in low- and middle-income countries with reduced access to sophisticated treatments. Furthermore, long-term follow-up studies with an isolated surgical approach to tricuspid valve disease, whether primary or secondary, do not show an impact on increased survival in 5 to 10 years, with improvement only in symptoms of right heart failure and quality of life.^9^ More recently, a randomized trial showed that concomitant tricuspid repair in patients with degenerative mitral regurgitation did not reduce mortality alone through a 2-year follow-up and was associated with more pacemaker implantations.^10^

This study, therefore, aims to evaluate the impact on surgical mortality of performing tricuspid repair concomitantly with mitral valve surgery in a population of patients from a developing country who have moderate to severe TR with advanced-stage disease undergoing cardiac surgery for rheumatic mitral valve disease.

## Methods

### Study Design and Patients

This is a prospective cohort study carried out from January 1, 2017, to December 30, 2022. All patients over 18 years of age who underwent cardiac surgery to correct rheumatic mitral valve disease with associated moderate-to-severe functional TR were included. All participants underwent a transthoracic echocardiogram performed 6 months before surgery and a second control echocardiogram performed after hospital discharge at a specialized hospital in northeastern Brazil. In this hospital, a monthly average of 100 cardiac surgeries are performed, with healthcare provided exclusively by the public health system. Patients with primary tricuspid valve involvement, active infectious endocarditis, and associated congenital heart disease were excluded. An exploratory analysis involving clinical and echocardiographic data with the aim of evaluating the long-term impact of tricuspid repair was also carried out 2 years after surgical procedure.

The diagnosis of rheumatic valve disease was made through the evaluation of clinical history and echocardiographic data. The presence of commissural fusion, chordal thickening, restricted leaflet motion, or a fixed posterior leaflet defines rheumatic involvement.^11^ Moderate to severe TR was diagnosed when there was the presence of a *vena contracta* ≥ 3 mm, an effective regurgitant orifice >0.2 cm^2^, or reverse systolic flow in the hepatic vein.^12^

The definition of the approach for TR occurred after a joint discussion between the clinical and surgical teams. All surgeries were performed using median sternotomy and the use of cardiopulmonary bypass (CPB) and an aortic cross clamp; tricuspid annuloplasty was performed using the DeVega or bovine pericardium patch implant techniques, according to the anatomy of the tricuspid ring during the operation.

This research was registered and followed the necessary procedures; the project was approved by the local ethics committee under the registration number CAAE 39774620.4.0000.0045.

### Outcomes

The primary outcome was surgical mortality, which is defined as in-hospital death (during hospitalization following the surgery) or on the first 30 days after the operation (in or out of hospital). Possible predictive variables were evaluated: symptoms by the New York Heart Association classification assessed at admission, right ventricular dysfunction, left ventricular ejection fraction, and systolic pulmonary artery pressure (sPAP) value, defined by echocardiogram evaluation. Additionally, clinical variables such as functional class and hospitalizations, as well as transthoracic echocardiographic data were obtained 2 years after the procedure.

### Statistical Analysis

For the statistical analysis, patients were divided into two groups according to tricuspid valve repair during surgery. The Kolmogorov-Smirnov test was used to verify the normal distribution of continuous variables. Variables with normal distribution were described by means and standard deviations, while those with nonsymmetrical distribution were described by medians and interquartile range. Categorical variables are described as frequency and percentage. Comparison groups were made using the t test for parametric distribution variables and the Mann-Whitney test for nonparametric distribution variables. Variables that showed a possible association in univariable analysis with the outcome of death (*p* < 0.1) or those with biological plausibility were included in the multivariable model through binary logistic regression. The probability value < 0.05 was considered statistically significant. The Statistical Package for the Social Sciences version 20.0 was used for data analysis.

## Results

Initially, 194 patients were evaluated, but, of these, 41 were excluded for having a nonrheumatic etiology for mitral dysfunction, 2 for having an associated interatrial septal defect, 5 for having primary rheumatic tricuspid valve disease, and 2 for having active endocarditis; thus, 144 patients were included in the study. Of these, only 83 (57.6%) underwent tricuspid valve annuloplasty.

The mean age was 46.2 (±12.3) years with 107 (74.3%) female individuals, the median left ventricular ejection fraction was 61.0% (55-67), and sPAP was 55.0 mmHg (46-74), with 45 (31.3%) individuals with right ventricular dysfunction. The predominant valve disease was mitral stenosis (74.3%). The group that was and the group that was not submitted to tricuspid repair had similar characteristics, except for the prevalence of severe TR, which was higher in the repair group: 65.1 vs. 47.5%; *p* = 0.001 (Table 1).

**Table 1:** Demographic and baseline clinical characteristics

Characteristic	Total (n = 144)	Tricuspid repair (n = 83)	No tricuspid repair (n = 61)	*p*∗
Age, mean (±SD)	46.2 (±12.3)	46.3 (±12.8)	46.0 (±11.5)	0.786
Female sex, n (%)	107 (74.3%)	65 (78.3%)	42 (68.9%)	0.199
Mitral stenosis moderate or severe, n (%)	107 (74.3%)	62 (74.7%)	45 (73.8%)	0.999
Mitral regurgitation moderate or severe, n (%)	83 (57.6%)	47 (56.6%)	36 (59.0%)	0.999
Severe tricuspid regurgitation, n (%)	83 (57.6%)	54 (65.1%)	29 (47.5%)	0.001
NYHA III or IV, n (%)	56 (37.5%)	32 (38.5%)	22 (36.0%)	0.865
Atrial fibrillation, n (%)	94 (65.3%)	56 (67.5%)	38 (62.3%)	0.519
LVEF, median (IQ)	61.0 (55.0-67.0)	62.0 (54.5-67.0)	60,00 (55.0-68.0)	0.886
sPAP, median (IQ)	55.0 (46.0-74.0)	57.0 (47.0-75.0)	51.00 (43.3-69.8)	0.124
Dysfunction of right ventricle, n (%)	45 (31.3%)	27 (32.5%)	18 (29.5%)	0.531

Abbreviations: LVEF, left ventricle ejection fraction; NYHA, New York Heart Association; sPAP, systolic pulmonary artery pressure.

∗ *p* value: comparison between tricuspid repair group and no tricuspid repair.

The time of CPB was longer in patients undergoing tricuspid repair surgery: 99 ​minutes (75-140) vs. 85 ​minutes (61-118); *p* = 0.04, with a similar frequency of concomitant aortic valve replacement: 26 (31.3%) vs. 27 (44.3%); *p* = 0.11, or reoperation (Table 2).

**Table 2:** Surgical characteristics of individuals

Characteristic	Total (n = 144)	Tricuspid repair (n = 83)	No tricuspid repair (n = 61)	*p*∗
Aortic valve replacement, n (%)	53 (36.8%)	26 (31.3%)	27 (44.3%)	0.112
Myocardial revascularization, n (%)	6 (4.2%)	4 (4.8%)	2 (3.3%)	0.999
Reoperation, n (%)	25 (17.4%)	15 (18.1%)	11 (18.0%)	0.999
Mechanical prosthesis, n (%)	112 (77.8%)	64 (77.1%)	48 (78.7%)	0.822
CPB time, min (IQ)	90 (68-125)	99 (75-140)	85 (61-118)	0.038
Aortic cross clamp, min (IQ)	80 (55-115)	80 (62-115)	75 (51-105)	0.082

Abbreviation: CPB, cardiopulmonary bypass.

∗ *p* value: comparison between tricuspid repair group and no tricuspid repair.

The total surgical mortality was 15 (10.4%) individuals, and there was no difference between the groups who were or were not submitted to tricuspid repair: 10 (12.0%) vs. 5 (7.5%); *p* = 0.46, respectively.

**Figure 1:**
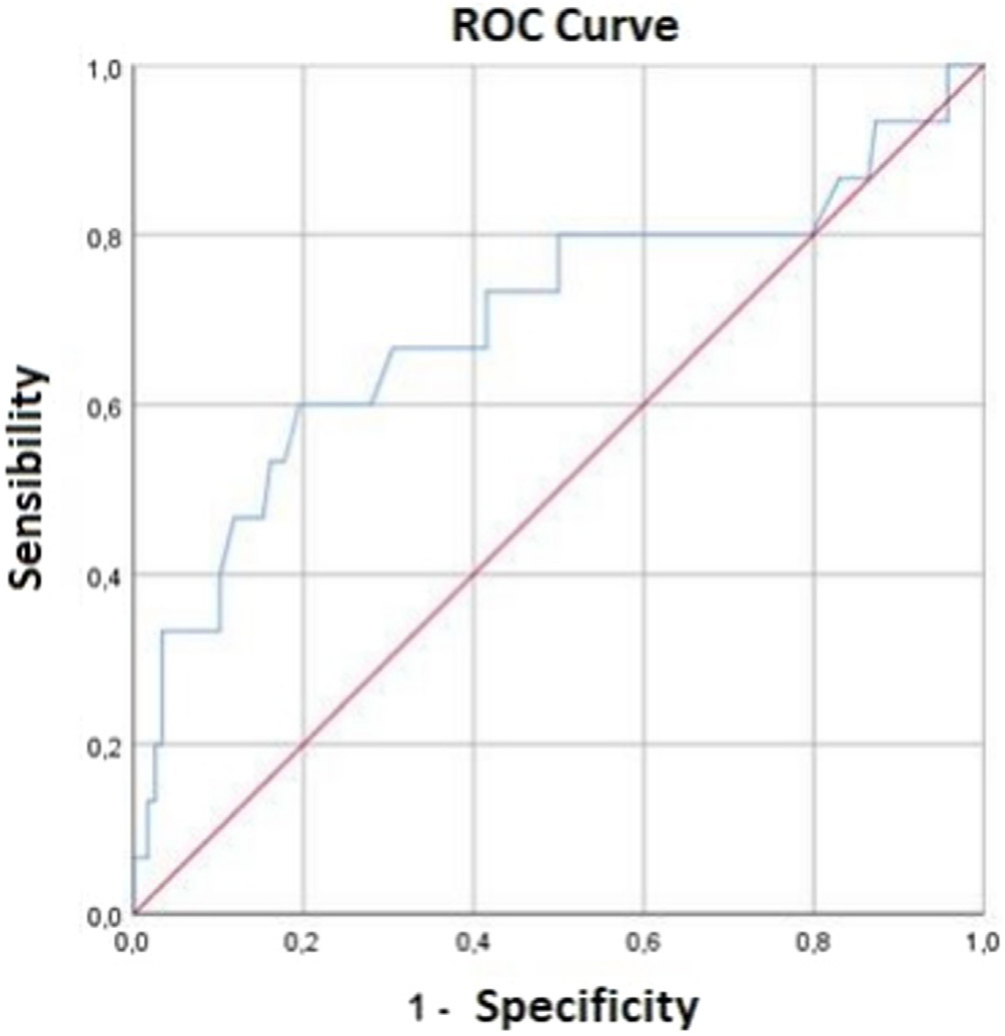
**ROC curve estimating 73.5 mmHg as the sPAP cut-off point with the highest accuracy for predicting surgical mortality and helping decision-making in clinical practice (area 0.70, *p* = 0.012)**. Abbreviation: ROC, receiver operating characteristic curve; sPAP, systolic pulmonary artery pressure.

In the multivariable analysis, involving four variables potentially predictive of death within 30 days, tricuspid repair had no association with death, relative risk 1.1 (0.3-3.9); *p* = 0.94. There was an association with one variable independently: the sPAP value, relative risk 1.04 (1.01-1.07), *p* = 0.01 (Table 3). To determine a sPAP cut-off that indicates higher mortality and can help decision-making in clinical practice, we performed an analysis through the receiver operating characteristic curve (area 0.70, *p* = 0.012) (Figure 1) which found that an estimated value of 73.5 mmHg has the highest accuracy in our model for predicting early mortality.

**Table 3:** Univariable and multivariable analysis for surgical death

Variable	Univariable analysis		Multivariable analysis	
	RR (95% CI)	*p*	RR (95% CI)	*p*
Age	1.01 (0.97-1.06)	0.599	1.02 (0.77-1.07)	0.319
Tricuspid repair	1.53 (0.50-4.74)	0.457	1.06 (0.29-3.89)	0.936
Severe TR	3.47 (1.05-11.49)	0.041	3.28 (0.87-12.30)	0.078
sPAP	1.04 (1.01-1.06)	0.002	1.04 (1.01-1.07)	0.003
NYHA III-IV	0.70 (0.22-2.23)	0.550		
Right ventricle dysfunction	1.62 (0.53-4.97)	0.403		
CPB time	1.01 (1.01-1.02)	0.001		
Aortic cross clamp time	1.01 (1.01-1.03)	0.003		
LVEF	1.04 (0.99-1.10)	0.109		

Abbreviations: CPB, cardiopulmonary bypass; LVEF, left ventricle ejection fraction; NYHA, New York Heart Association; RR, relative risk; sPAP, systolic pulmonary artery pressure; TR, tricuspid regurgitation.

In the exploratory analysis carried out 2 years after surgery, 81 (56%) patients were recalled with a mean time of 2.75 (±1.13) years. There was no difference between the groups who were or were not submitted to tricuspid repair regarding clinical outcomes such as repeat hospitalizations and functional class (Table 4).

**Table 4:** Clinical and echocardiographic outcomes (2 years after surgery)

Outcome	Total (n = 81)	Tricuspid repair (n = 44)	No tricuspid repair (n = 37)	*p*∗
Moderate to severe TR, n (%)	20 (24.7%)	7 (15.9%)	13 (35.1%)	0.051
NYHA III or IV, n (%)	3 (3.7%)	1 (2.3%)	2 (5.4%)	0.999
Hospitalization, n (%)	13 (16.1%)	9 (20.1%)	4 (10.8%)	0.209
RV diameter, median (IQ)	32.0 (29.0-36.0)	32.0 (29.0-37.3)	32.5 (29.0-36.0)	0.994
RA volume, mean (±SD)	65.5 (±31.9)	54.1 (±24.6)	78.2 (±34.8)	0.005
sPAP, median (IQ)	30.0 (25.0-36.0)	29.0 (23.3-35.0)	32.0 (27.0-37.0)	0.300
FAC (RV), mean (±SD)	43.8 (±9.3)	42.9 (±8.1)	44.8 (±10.6)	0.496

Abbreviations: FAC, fractional area change; NYHA, New York Heart Association; RA, right atrium; RV, right ventricle; sPAP, systolic pulmonary artery pressure; TR, tricuspid regurgitation.

∗ *p* value: comparison between tricuspid repair group and no tricuspid repair.

The control transthoracic echocardiogram showed moderate or severe TR in 7 (15.9%) patients who underwent tricuspid valve repair vs. 13 (31.5%) of conservative group; *p* = 0.05. There was a difference regarding the right atrium volume in the tricuspid repair vs. conservative treatment group during this follow-up period: 54.1 ml (±24.6) vs. 78.2 ml (±34.8); *p* = 0.01, however, there was no difference in right ventricular diameter and right ventricular systolic function evaluated through fractional area change method: 32 mm (29-37) vs. 33 mm (29-36); *p* = 0.99 and 43% (±8.1) vs. 45% (±11.0); *p* = 0.50, respectively.

## Discussion

In our study, tricuspid valve repair in patients with moderate or severe functional TR during the surgical approach to left-sided valve disease in individuals with rheumatic heart disease did not alter mortality within 30 days. This occurred despite a higher CPB time in the group undergoing tricuspid repair.

The mortality observed in our study was high, but our population consisted of high-risk individuals; more than a third of them also underwent aortic valve replacement (36.8%), and the prevalence of pulmonary hypertension was very high (86.1%), with a median sPAP of 55 mmHg and 31.3% also exhibiting right ventricle dysfunction. Thus, this procedure’s safety is reinforced even for a specific profile of advanced disease associated with overload of right chambers and a high prevalence of right ventricular systolic dysfunction. In addition, similar data regarding mortality were seen in other trials with a comparable population profile. Kuwaki et al.^13^ had an in-hospital mortality of 8.9% in surgeries for correction of left valve disease associated with significant tricuspid insufficiency in a Japanese center. Moreover, mortality records from cardiac surgery in low- and middle-income countries in this scenario are often elevated. Mejia et al., at a specialized Brazilian center in which many individuals are diagnosed and treated in late stages of the disease, described a surgical mortality of 8.9%.^14^ In this context, our study shows that tricuspid repair is not associated with increased mortality, even in a population from a developing country with an advanced stage of the disease.

Concerning the proportion of tricuspid repair performed according to the degree of the tricuspid disease, 81.5% of patients with severe TR underwent repair surgery, whereas of patients with moderate TR, only 38.1% underwent repair. These data are similar to those from a North American registry in which, despite the recommendations of the main guidelines, tricuspid annuloplasty was performed in only 79% of patients with severe TR and 39% of patients with mild to moderate TR during mitral valve intervention.^15^ These findings reinforce the view that moderate tricuspid valve dysfunction is underestimated in the context of the left valve approach.

After a multivariable analysis, the value of sPAP was identified as predictor of higher surgical mortality. Individuals with greater severity of pulmonary hypertension are more likely to develop acute right ventricular dysfunction and circulatory shock in the immediate postoperative period.^16^^,^^17^ The association between pulmonary hypertension and surgical death is already described in the literature; however, a cut-off point of sPAP that can discriminate individuals at higher and lower risk is not well established. In our model, values of sPAP above 73.5 mmHg were shown to predict a higher risk.

The results of the present study suggest the safety of tricuspid repair, reinforcing the recommendations in the guidelines for valvular heart disease. However, the severity of pulmonary hypertension should be considered in the discussions of the heart team when dealing with patients with a natural history of advanced disease and high surgical risk in weighing risks vs. benefits of left valve surgery.

In view of previous studies demonstrating that there is a reduction in symptoms of right heart failure and improvements in quality of life,^9^ we continued the long-term follow-up of these individuals to assess the echocardiographic data of right ventricular function related to tricuspid repair. There was no difference regarding function and diameter of right ventricle during a mean follow-up time of 2.75 years. There was a difference, however, in right atrium volume favoring the tricuspid repair group. Regarding functional class, a longer period of follow-up would probably be necessary to notice a difference between the two groups, as previous studies show that patients with previous dilation of the tricuspid ring are at greater risk of developing TR progression and worse outcomes.^18^

This study has limitations: it is a single-center cohort, subject to the biases inherent to this model of study. The sample size was low, and the fact that there is no difference between the groups may be related to a type 2 error (lack of power), but the study maintains its clinical relevance because the population studied is composed exclusively of patients with rheumatic mitral valve disease. This reduces the heterogeneity and enhances the study's clinical importance since there is a paucity of studies contemplating this particular population in this particular scenario. Long-term clinical variables were analyzed despite considerable loss of follow-up (44%). This analysis, therefore, suffers from a lack of power and is exploratory in nature. A longer follow-up period would also be necessary to detect a relevant difference between the two groups. Despite these limitations, with this small sample, it was already possible to observe a smaller volume of the right atrium in the follow-up of patients who underwent tricuspid repair.

## Conclusions

Performing tricuspid repair in individuals who had moderate to severe tricuspid insufficiency was not associated with increased surgical mortality in patients from a developing country undergoing cardiac surgery for rheumatic mitral valve disease. This result reinforces the recommendation of current guidelines that the procedure is safe in this specific high-risk population. sPAP value was independently associated with surgical death.

## Ethics Statement

The present study was approved by the ethics committee of the institution where it was carried out (registration number: CAAE 39774620.4.0000.0045). All the procedures were conducted in accordance with the Declaration of Helsinki.

## Consent Statement

All patients provided written informed consent.

## Funding

The authors have no funding to report.

## Disclosure Statement

The authors report no conflict of interest.
